# The Effects of Ageing on Intergenerational Support Exchange: A New Look at the Hypothesis of Flow Reversal

**DOI:** 10.1007/s10680-018-9472-6

**Published:** 2018-04-09

**Authors:** Matthijs Kalmijn

**Affiliations:** 1 Department of Sociology, University of Amsterdam, Nieuwe Achtergracht 166, 1018 WV Amsterdam, The Netherlands; 2 Netherlands Interdisciplinary Demographic Institute (NIDI), The Hague, The Netherlands

**Keywords:** Intergenerational relationships, Ageing, Life course, Family, Social exchange

## Abstract

There has been debate about whether the flow of intergenerational support reverses as parents age. One view is that in western countries, parents remain ‘net donors’ to children, even in very old age. Such a conclusion coincides with notions of parental altruism and would be in contrast to notions of exchange and reciprocity over the life course. This paper examines the thesis of flow reversal in a new way: it uses prospective longitudinal data, it combines data from samples of ageing parents and samples of adult children, it develops a way to create measures of balance from frequency items on support exchange, and it combines objective measures of support exchange with subjective perceptions of symmetry. The focus is limited to support that involves time and effort. The support that parents give to children declines with age, the support they receive increases, and at around age 75–76, parents become ‘net receivers’. The decline in downward support is stronger than the increase in upward support, suggesting that declining parental opportunities to give plays an important role in the flow reversal. In sum, the analyses provide evidence for what we can call delayed and parent-driven flow reversal. Evidence for flow reversal is stronger in the sample of adult children, pointing to the limitations of sampling ageing parents. Finally, there is correspondence between objective measures of support exchange and perceptions of symmetry, although on the whole, few parents regard themselves as ‘net receivers’.

## Introduction

A common hypothesis in the literature on intergenerational relationships is that the flow of support reverses as parents age (Rossi and Rossi 1990). When children are young adults, parents would give more support to children than they receive, but when parents become older, the support that children give to parents would increase and the support that parents give would decline, turning parents from ‘net donors’ into ‘net receivers’. There are several reasons why such a flow reversal has been assumed. First, the demand for support among parents increases as they age. Older parents have more health problems, their network becomes smaller, and they are at risk of losing a partner through divorce or death. Second, the opportunities for giving support decline with age. Older parents are less mobile, less fit physically, and ill more often, and this makes it more difficult for them to give support to their children. Third, the demand for support among children declines as children become older. When children are making the transition to adulthood, they need various types of support, such as help in setting up a new household, advice and guidance in making important life decisions, and support in raising and caring for their children. When children are older—which is also when parents are older—children’s demand for support declines.

That the flow of support reverses at some age is in line with more general ideas about social exchange and extended or delayed reciprocity in intergenerational relationships (Attias-Donfut 2003; Silverstein et al. 2002; Kalmijn 2014). Delayed exchange is difficult to test directly because there can also be immediate exchange (Leopold and Raab 2011). Nonetheless, long-term longitudinal studies of support in intergenerational relationships tend to confirm the principle of delayed exchange (Silverstein et al. 2002) and studies of transfer motives provide positive evidence as well. In the German ageing study, for example, 78% of respondents agreed with the statement that ‘my parents have done so much for me that I would like to give them back some of it’ (Kohli and Künemund 2003).

The hypothesis of flow reversal is well known, but it has also been criticized (Albertini et al. 2007; Litwin et al. 2008; Grundy 2005). An obvious argument against flow reversal lies in financial support. Albertini et al. (2007) and Litwin et al. (2008), for example, argue that the net flow tends to remain downward because parents often make financial transfers to children, especially when they are older. Flow reversal may also fail to occur because children’s demand for support may not decline over the life course. Children may become unemployed, they may separate, move house, and all such later life course transitions go hand in hand with a persisting need for practical and emotional support from parents (Seltzer et al. 2012; Timonen et al. 2011). Another criticism comes from the theory of developmental stake which argues that children are often more important emotionally to parents than parents are to children (Birditt et al. 2015; Mandemakers and Dykstra 2008). As a result, parents may continue to invest in their children, even when the children themselves are older. In a similar fashion, theories about intergenerational ambivalence have argued that parents sometimes have difficulties in accepting the child’s autonomy as children become adults, just as children may tend to cling on to the role of a dependent child (Luescher 2002; Fingerman et al. 2011). The difficulty to ‘switch roles’ during the life course can lead to a continued stream of support from older parents to middle-aged children. Such a pattern coincides with notions of parental altruism and would be in contrast to notions of exchange and reciprocity over the life course.

Although it is difficult to test the hypothesis of flow reversal conclusively, a closer look at the effects of parental age on support exchange is an essential part of this test. Although there are many studies on support exchange between adult children and their elderly parents, the number of studies that have specifically isolated the effects of parental age is limited. Moreover, the studies that have been done are based on a cross-sectional design rather than a longitudinal design. In an analysis of older parents in Boston in the 1980s, Rossi and Rossi (1990) found declines in the support that parents gave with age but—surprisingly—also declines in the support that they received. Because the former decline was quicker than the latter, there was a reversal of the net flow, estimated at age 70. In an analysis of older parents in Britain in the late 1980s and early 1990s, Grundy (2005) found that parents in all age groups gave more than they received, thereby refuting the hypothesis of flow reversal altogether (Grundy 2005). In an analysis of the 2004 wave of the SHARE data on instrumental support, Albertini et al. (2007) found that the balance of intergenerational time transfers became more favourable to parents with age; even at age 70, however, parents were more often net donors than net receivers.

## Research Question and Contribution

In the present paper, I use the *Netherlands Kinship Panel Study* to re-examine how the exchange of support changes as parents age (Dykstra et al. 2005). The focus is on practical, social, and emotional support and not on financial support. Several problems in the study of intergenerational balance are addressed. First, I analyse prospective longitudinal data rather than cross-sectional data. Most estimates of age effects on support have been based on cross-sectional designs and are potentially biased by the confounding effects of birth cohort. The present paper examines the effects of parental age on receiving and giving support based on a prospective longitudinal data set and fixed-effects regression models that compare changes in the flow of support within dyads as parents age (Allison 2009). The aim is to describe age effects in detail, not to explain age effects using mediating characteristics of parents and children.

Second, analyses are presented from the perspective of both the child and the parent (albeit not from parents and children who are related to each other). I analyse respondents in their role of ageing parents (reporting on two of their children) and respondents in their role of adult children (reporting on their ageing mothers and fathers). This addresses two well-known issues in the literature. Older people with health problems are often underrepresented in surveys, and the age range of parents in a set of ‘child’ data is much wider than it is in a set of ‘parent’ data (Hardy et al. 2009; Chatfield et al. 2005; Lundberg and Thorslund 1996; Kelfve et al. 2013). In addition, several studies have shown that family members overestimate giving and underestimate receiving (Klein Ikkink et al. 1999; Walker et al. 1991; Lin 2008; Kim et al. 2011; Attias-Donfut 2003). Discrepancies can partly be systematic but will also be caused by measurement error. In either case, they call for combining the two perspectives and finding a middle ground between reports of parents and children.

Third, I develop measures of balance where different types of support and different frequencies of support exchange are scaled based on an empirical method. A well-known problem in analysing balance lies in the fact that streams of different types of support cannot be compared very well. To solve this, some authors have used a disaggregated approach where each type of support in an upward direction is compared to the same type of support in a downward direction (e.g. Grundy 2005; Rossi and Rossi 1990). A disadvantage is that this approach does not yield an overall measure of balance. An alternative is to use time (hourly) measures of upward and downward support (Albertini et al. 2007). While this leads to the desired summary measures of balance, many surveys do not have time measures. Moreover, time measures do not cover the more social and emotional aspects of support. Developing measures of balance is a difficult task which always rests upon a set of assumptions. In this paper, I try to develop another solution by empirically scaling the different types of support.

Fourth, I examine not only the actual levels of support but also how parents and children *perceive* the degree of symmetry in the relationship. Previous studies have shown that relationship quality and conflict in parent–child dyads are affected by both subjective and objective measures of balance; hence, it is important to examine both aspects in one study (Sechrist et al. 2014; Wahrendorf et al. 2010). More importantly, it is possible to relate these subjective measures of symmetry to the behavioural scales of the balance of support. These analyses can show to what extent objective measures come back in the views that parents and children themselves have about the relationship. This analysis does not address the reliability of the support measures, but it does address their validity. If objective and subjective measures are not related empirically, this would throw doubts about the given approach.

The context of this study is the Netherlands, which belongs to the western European cluster of countries in terms of intergenerational support and contact (Hank 2007; Albertini and Kohli 2013) but that is also on the individualistic side in terms of its culture (Hagenaars et al. 2003). Norms to support parents do exist but very few people believe that children should take older parents in their home to care for them (De Vries et al. 2009). Population ageing is perceived as a societal problem, and government policies increasingly emphasize the importance of informal solutions in meeting the increasing demand for support and care in the population of older adults (De Boer 2006; De Klerk 2011). The increase in the number of older adults who live independently rather than in institutional care homes further contributed to the increase in demand for informal care from family members (Garssen 2011).

## Method

### Data

Four waves of data have been collected in the Netherlands Kinship Panel Study (Merz et al. 2012; Hogerbrugge et al. 2015; Dykstra et al. 2005, 2007). The survey was based on a nationally representative sample of adult individuals in the Netherlands. Data were collected in four waves starting in 2002/2003. Each wave was collected 4 years after the previous wave so that the total period covered is 12 years. The number of respondents was 8161 in the first wave, and wave-to-wave panel continuation rates were 75, 72, and 65%, respectively. For an interval of 4 years between waves, this is reasonable although it is lower than what has been achieved in annual panel surveys in the USA, the UK, and Germany. I have replicated all (random effects) models using weights to correct for sample attrition. Weights were based on probit models for staying in the sample between waves (Vandecasteele and Debels 2007). Since weighted and unweighted models yielded similar effects, I abstain from correcting for sample attrition in the main tables. My starting sample consists of respondents who participated in at least the first two waves (*n* = 6091). Descriptive information on variables and samples is included in Table 1.

**Table 1:** Descriptive statistics of parent and adult child samples

	Mean	SD	Min	Max	Count
Ageing parent sample					
Age child	35.341	7.078	21	58	9712
Age parent	66.952	7.414	55	90	9715
Child to parent	19.224	9.607	0	46	9715
Parent to child	22.302	10.690	0	46	9715
Parent to child+	27.070	15.627	0	64	7021
Up–down	− 3.077	11.196	− 46	45	9715
Up–down+	− 7.885	15.241	− 64	45	7021
Up–down (perceived)	− .502	1.291	− 3	3	8131
Mother versus father	.001	.493	− 1	0	9715
Daughter versus son	− .065	.500	− 1	0	9715
Parent higher education	.036	.456	− 0	1	9715
Child higher education	− .007	.492	− 0	1	9715
Adult child sample					
Age child	41.925	8.919	21	76	15,698
Age parent	71.189	9.608	55	103	15,698
Child to parent	23.323	11.670	0	46	15,697
Parent to child	19.622	10.456	0	46	15,697
Parent to child+	22.714	14.285	0	64	15,697
Up–down	3.701	12.779	− 46	46	15,697
Up–down+	.609	15.836	− 64	46	15,697
Up–down (perceived)	.165	1.587	− 3	3	13,346
Mother versus father	.000	.493	− 1	0	15,698
Daughter versus son	.039	.486	− 1	0	15,698
Parent higher education	− .024	.424	− 0	1	15,684
Child higher education	.009	.495	− 0	1	15,698

Parent to child+ and up/down+ include grandparenting

*Source*: Netherlands Kinship Panel Study

The NKPS interview included an elaborate support module in which support exchange was assessed for a range of family members, including the biological father and mother and two randomly chosen children age 15+. Based on this module, two subsamples were constructed. For the first sample (‘the parent sample’), I selected men and women who were 55 or over during the panel with at least one child 21+ who was not living with the respondent (in the first wave). Persons with missing values on intergenerational contact were excluded (less than 1% of the cases). This yields a sample of 3440 parent–child dyads. For the second, ‘adult child sample’, I selected men and women who were 21+ and who had at least one mother or father of 55 or older during the panel (and who was still alive in the first two waves). This yields a sample of 5541 parent–child dyads. Note that some of the dyads can be in both samples. The parent–child dyad is the unit in my analyses (*N*_dyads_ = 8981). Each dyad has a unique id that is used in the fixed-effects model, but the standard errors are adjusted for the clustering of dyads in families (*N*_families_ = 5145).

### Measures

The following types of help were assessed: (a) support with household work, (b) support with (other) practical matters, (c) giving or receiving (good) advice, (d) showing an interest in the parent’s or child’s personal life (a measure of emotional support), and (e) helping to take care of grandchildren (only in a downward direction). For each type of help, we asked how often it was given or received in the past 3 months. Answers could be ‘never’, ‘once or twice’, or ‘more often’. Note that in the first wave, the question on grandparenting was only asked among children.

Because there were no time measures available—such measures are rare in most surveys—a scaling procedure was developed. This procedure needed to address (a) how different categories within an item can be compared and (b) how similar categories across support items can be compared. To solve this, an auxiliary regression model was developed in which the annual contact frequencies between parents and children (measured in detail) were regressed in a multivariate model on each type of support. Each type of support was coded using two binary variables with no support as a reference category (see Appendix Table 7). The regression models show how the levels of contact differ between different types and levels of support. For example, the difference between ‘no’ household support and ‘incidental’ support is smaller than the difference between ‘incidental’ and ‘often’ household support and ‘often’ means more frequent contact for grandparenting than for practical support. The regression estimates can be used to scale the variables. Specifically, the predicted numbers of contacts based on these regression models were used to scale each category of each support variable (see Appendix Table 7). Of course, such a category value does not pertain to each individual in that category, but it is on average close to the amount of contact that was used to provide or receive that specific type and level of support.

Using the five scaled support items, I construct three dependent variables: the sum of support given to the child, the sum of support received from the child, and the difference (received minus given). The scale for downward support includes five items, and the scale for upward support includes four items (grandparenting is not included). I will show empirically how including grandparenting affects the age effects. Specifically, I compare two measures of downward support (with and without grandparenting) and the two corresponding measures of balance. For upward support, there is only one measure.

We also asked respondents how they perceived the balance of support. The question asked by the interviewer was as follows: ‘Giving and receiving is an important aspect of relationships. How would you describe your relation with [name, description], do both of you give about the same amount, do you give more than the other, or does the other give more than you?’ The answering categories were (1) respondent gives more, (2) both give about the same, and (3) the other gives more. It needs to be recognized that in evaluating balance, parents and children may include past exchanges. To the extent that they do, there will be less correspondence between perceived balance and the actual balance of support at the time of the survey. The question was not asked in the fourth wave.

### Models

Fixed-effects regression models were used to assess the effects of parental age on the objective measures of support. I include parent’s age and parent’s age squared as independent variables. Since parental age is an exogenous variable, there is no need to consider confounding variables. The effects of parental age can be mediated by other changes and life events, but the aim was to describe the effects of parental age, not to explain these effects. I present both linear and quadratic models to evaluate if the age patterns are linear. Age is centred around 70 for parents. This specification facilitates the interpretation of the main effects of age in the quadratic model: they reflect the age slope at age 70. Centring does not affect the linear model for age. (It only affects the intercept in that model.)

To estimate if and if so, at what age the flow of support reverses, I present specific estimates. In the model where the balance variable (support received minus given) is the dependent variable, I estimate the age at which the expected value of the dependent variable is 0. Assuming that there is an upward slope, this is the age at which the parents turn from ‘net donors’ (negative values) into ‘net receivers’ (positive values). If the fixed-effects model is defined for individual *i* at time point *t* as follows: *Y*_*it*_ = *b*_0_ + *b*_1_ AGE_*it*_ + *e*_*t*_ + *e*_*i*_, the crossing point is implied at AGE_*it*_ = − *b*_0_/*b*_1_. If the model is defined as: *Y*_*it*_ = *b*_0_ + *b*_1_ AGE_*it*_ + *b*_2_ AGE_*it*_ * AGE_*it*_ + *e*_*t*_ + *e*_*i*_, the crossing point is implied at AGE_*it*_ = [− *b*_1_ + √(*b*_1_^2^ – 4 *b*_2_*b*_0_)]/[2 *b*_2_] or AGE_*it*_ = [−*b*_1_ − √(*b*_1_^2^ − 4 *b*_2_*b*_0_)]/[2 *b*_2_].

I also examine how the age patterns differ between men and women and between educational groups. Given the very different levels of contact and proximity in parent–child ties between classes or educational groups (Kalmijn 2006; Shelton and Grundy 2000) and between men and women (Kalmijn 2007; Clark and Kenney 2010; Rossi and Rossi 1990; Haberkern et al. 2015; Attias-Donfut 2003), this is an important issue to explore. Moderator effects of gender and education are examined separately. Education is based on both parents and children and coded as a dichotomy (1 for higher vocational or university education and 0 otherwise). In the children sample, I used information on father’s education only since many mothers in this generation were not highly educated. Interaction effects are calculated separately for the child’s and the parent’s education. Interactions by gender of parent and child are also included. Random-effects models are used rather than fixed-effects models because this makes it possible to look at the main effects of education and gender as well.

## Findings

### Actual Exchange in Ageing Parent Data

Results for models where parents are the respondents are presented in Table 2. The fixed-effects model shows that the support that parents receive increases significantly with age. The quadratic specification suggests that this increase becomes steeper with age. In the second model of Table 2, we see that the support that parents give to children declines significantly with age. This effect is stronger when adding grandparenting to the scale of downward support. The effect of parental age is stronger for how much they give (− .27 and − .49) than for how much they receive (.07).

**Table 2:** Fixed-effects models of age effects: parent reports

	(1)	(2)	(3)	(4)	(5)
	Child to parent	Parent to child	Parent to child+	Up–down	Up–down+
Age parent (− 70)	.067*	− .274*	− .493*	.341*	.581*
	(.028)	(.030)	(.064)	(.035)	(.069)
Constant	19.430*	21.468*	25.989*	− 2.038*	− 6.609*
	(.086)	(.092)	(.141)	(.107)	(.151)
Reversal age				*76.0*	*81.4*
Dyads × waves	9715	9715	7021	9715	7021
Dyads	3440	3440	3418	3440	3418
*F*	5.68	81.41	58.93	94.13	70.98
S.d. between	8.098	8.491	13.023	8.206	12.245
S.d. within	7.030	7.930	10.891	9.098	11.561
Age parent (− 70)	.124*	− .319*	− .617*	.443*	.751*
	(.032)	(.030)	(.062)	(.037)	(.069)
Age squared	.011*	− .009*	− .027*	.020*	.037*
	(.002)	(.002)	(.004)	(.003)	(.005)
Constant	18.895*	21.898*	27.357*	− 3.003*	− 8.477*
	(.135)	(.155)	(.273)	(.170)	(.300)
Reversal age				*75.5*	*78.1*
Dyads × waves	9715	9715	7021	9715	7021
Dyads	3440	3440	3418	3440	3418
*F*	12.69	56.82	64.86	76.91	71.77
S.d. between	8.074	8.489	12.999	8.157	12.140
S.d. within	7.030	7.930	10.891	9.098	11.561

Standard errors in parentheses. Parent to child+ and up/down+ include grandparenting

^~^*p* < .10; *p < .05

*Source*: Netherlands Kinship Panel Study

When analysing the balance of support directly, we see clear positive age effects, which is logical given that the effect on receiving from children is positive while the effect on giving to children is negative. At what point does the balance change? I use the better fitting quadratic estimates to estimate the point at which the level of balance crosses the 0-line. Grandparenting is included in the measure. The point at which the estimated level of balance becomes 0 is 78.1. After this age parents turn from net donors into net receivers. This result suggests that flow reversal does occur but rather late in the parent’s life course.

### Actual Exchange in Adult Children Data

The results just discussed are based on the reports of ageing parents in the NKPS. In Table 3, I present parallel results when the respondent is the adult child. I first find that the support that parents receive increases with age. We also see that the support that parents give declines with age. Together, these two age patterns result in a positive age effect on the balance of support. As is found in Table 2, the effects of parental age on downward support and on the balance of support are stronger when grandparenting is included.

**Table 3:** Fixed-effects models of age effects: adult child reports

	(1)	(2)	(3)	(4)	(5)
	Child to parent	Parent to child	Parent to child+	Up–down	Up–down+
Age parent (− 70)	.375*	− .139*	− .230*	.514*	.605*
	(.026)	(.021)	(.030)	(.029)	(.036)
Constant	22.877*	19.788*	22.987*	3.089*	− .111*
	(.031)	(.025)	(.036)	(.035)	(.043)
Reversal age				*64.0*	*70.2*
Dyads × waves	15,697	15,697	15,697	15,697	15,697
Dyads	5541	5541	5541	5541	5541
*F*	208.42	44.49	59.09	312.39	279.86
S.d. between	9.600	8.804	11.913	8.884	11.142
S.d. within	8.010	6.838	9.068	9.280	10.967
Age parent (− 70)	.320*	− .132*	− .200*	.453*	.520*
	(.025)	(.022)	(.032)	(.028)	(.036)
Age squared	.015*	− .002	− .008*	.017*	.023*
	(.001)	(.001)	(.002)	(.002)	(.002)
Constant	21.533*	19.964*	23.723*	1.568*	− 2.190*
	(.134)	(.107)	(.139)	(.155)	(.179)
Reversal age				*65.9*	*73.6*
Dyads × waves	15,697	15,697	15,697	15,697	15,697
Dyads	5541	5541	5541	5541	5541
*F*	154.38	25.63	64.39	203.59	243.27
S.d. between	9.595	8.802	11.916	8.856	11.144
S.d. within	7.943	6.837	9.051	9.206	10.850

Standard errors in parentheses. Parent to child+ and up/down+ include grandparenting

^~^*p* < .10; *p < .05

*Source*: Netherlands Kinship Panel Study

While the effects are similar to the results for the parent data in Table 2, there are differences in the strength of the effects and these have important consequences for the overall conclusion. The positive age effect on the support that parents receive is stronger when children report than when parents report. Because the effects are different in strength, the point at which the flow reverses is located at an earlier age in the adult child data than in the parent data. In the adult child data, the flow of support reverses when parents are 73.6.

**Fig. 1:**
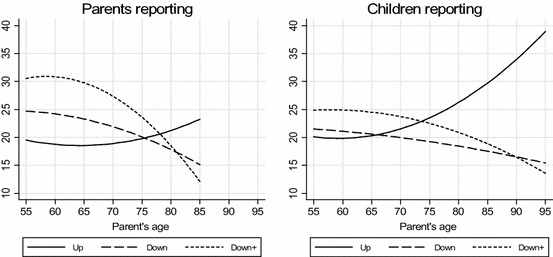
Predicted parental age patterns of support in child and parent samples

In Fig. 1, I illustrate the age patterns based on the fixed-effects models in the parent data (left side) and the adult child data (right side). We see age-related declines in downward support, but this decline is stronger in the parent data. We see age-related increases in upward support, but this increase in parents’ receiving is stronger in the adult child data. The age range is wider when children report and we see much ‘action’ in that last period, especially a considerable increase in the amount of support that parents receive. If this late increase is missed by design, as is the case in the parent data, there is a risk of underestimating the age effect and this may result in a later age at reversal. This problem is probably due to the fact that ageing parents with more extreme health problems are underrepresented in the parent data but not in the adult child data. At very high ages, we now see considerably more upward than downward support, something that was not visible in the parent data. Also clear is that the age at which the lines cross (where upward and downward support are equal) is located earlier in the child data. Finally, when I include grandparenting (‘parent to child+’ in the graph), the overall level of downward support is higher, which leads to a later crossing point and hence, a later age at reversal.

### Combing Parent and Child Data

Which design should we prefer? The child sample will contain higher numbers of very old and frail parents, and this would motivate a choice for the adult child design. Yet children may over-report what they are giving to their parents, something that would be consistent with a more general tendency to overstate one’s own contributions in personal relationships (Kamo 2000; Tao 2013). If this bias occurs, it would not be wise to rely solely on the adult child data.

For these reasons, it is useful to find a middle ground. To accomplish this, I pooled the data and estimated the age effects in the pooled data. The two samples are represented equally using a weighting scheme. This ensures that the estimates are ‘halfway’. Results are presented in Table 4. In the pooled data, I find a parental age effect on upward support of *b* = .221 and a parental age effect on downward support of *b* = − .309. Hence, the decline in downward support is stronger than the increase in upward support. The effect on the balance of support is *b* = .598 and the crossing age is located between ages 75 and 76. In other words, if ‘the truth is in the middle’, there is some support for the hypothesis of flow reversal. Giving to children declines, receiving from children increases, and after age 75–76, parents become net receivers. Current life expectancy in the Netherlands (for 55-year olds) is 82 for men and 85 for women (www.statline.cbs.nl) and continues to increase. Hence, the period during which parents are net receivers is not short.

**Table 4:** Fixed-effects models of parental age effects using pooled weighted data

Dependent variable	Without grandparenting		With grandparenting	
Up: from children to parents	.221	(.014)	.268	(.017)
Down+: from parents to children	− .309	(.021)	− .328	(.022)
Balance	.598	(.025)	.596	(.025)
Age at reversal (linear model)	*74.7*		*75.6*	
Age at reversal (quadratic model)			*75.9*	
*N*	25,412		22,718	

Parent and child sample weighted equally in each model. Standard errors in parentheses

*Source*: Netherlands Kinship Panel Study

A final question that arises is how strong the effects are. Effects are significant, but the samples are large so statistical significance alone is a poor guide. To explore this, I calculate standardized regression coefficients in a linear regression model. The beta’s for parental age are + .19 for upward support, − .33 for downward support, and + .44 for the balance variable. These estimates show that the effects of parental age are quite strong. Note that the strength of the effect is based on the variance both between dyads and within dyads over time.

### Comparisons with Perceived Balance

**Fig. 2:**
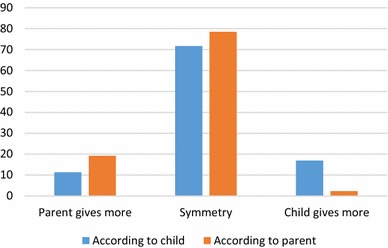
Perceived symmetry in relationship in parent and child samples

To what extent is my objective measure of support reflected in the way parents and children perceive the flow of support? First, it is instructive to look at the frequencies of the perceived symmetry variable, as presented in Fig. 2. We see that the large majority of parents and adult children say that the relationship is more or less symmetrical. Perhaps the use of only three categories in the question has resulted in a relatively large symmetrical group. The share of children who say that they are giving more is larger than the share of children who say that they are receiving more. When looking at parents, the results are different. There are more parents who say they give more than there are parents who say that they receive more. Perhaps this can be explained by the fact that older parents are uncomfortable with being dependent on their children. It is also possible that parents incorporate past transfers to children in their answers. Moreover, the question on perceived symmetry does not exclusively apply to the support dimensions that are measured in the NKPS. Less tangible forms of support and financial support may be missed in these measures but can be important for how parents and children evaluate their relationship.

A more important issue is how the measure of perceived balance is related to age and to objective measures of support. The subjective measure was analysed with a random-effects multinomial logistic regression model where symmetry served as the base category (Table 5). In the top panel, parents are the ones who reported, in the bottom panel, adult children are the ones who reported. The *N* is lower than in the analyses of support exchange because the question was not asked in the fourth wave. The table shows that when parents report, parental age has a positive effect on being a net receiver and a negative effect on being a net donor. In the adult child sample, I find that as parents age, children believe they are net donors more often and net receivers less often. In other words, the age effects in the two samples are similar, despite the fact that the frequency distributions are different in the two samples. More importantly, the age effects are in line with what was found for the objective measure of support balance.

**Table 5:** Random-effects multinomial logit regression for the effects of age and support exchange on perceived balance

	Parent reports		Parent reports		Child reports		Child reports	
	Model 1		Model 2		Model 1		Model 2	
	Parent gives more	Child gives more	Parent gives more	Child gives more	Parent gives more	Child gives more	Parent gives more	Child gives more
Age parent (− 70)	− .0385*	.0729*	− .0258*	.0555*	− .0731*	.0972*	− .0409*	.0323*
	(.0064)	(.0122)	(.0078)	(.0162)	(.0055)	(.0048)	(.0056)	(.0045)
Balance household support			− .0104	.0950*			− .0300*	.0361*
			(.0096)	(.0220)			(.0066)	(.0064)
Balance practical support			− .0323*	.0305			− .0431*	.0599*
			(.0120)	(.0261)			(.0088)	(.0086)
Balance emotional support			− .1368*	.0100			− .0654*	.1417*
			(.0128)	(.0290)			(.0113)	(.0081)
Balance advisory support			− .1679*	.1769*			− .1194*	.2102*
			(.0233)	(.0507)			(.0179)	(.0163)
Grandparenting			.0029	− .0308			.0337*	− .0674*
			(.0070)	(.0188)			(.0057)	(.0086)
Constant	− 2.3665*	− 4.6301*	− 2.6200*	− 5.0230*	− 3.0886*	− 2.6603*	− 3.0860*	− 2.6693*
	(.0808)	(.2607)	(.1237)	(.4830)	(.0917)	(.0741)	(.0931)	(.0748)
Respondent × waves	8131		5437		13,346		13,345	
Log likelihood	4495		3006		9301		8672	

In the second model, objective measures of support are added to evaluate how objective and subjective measures align. I include the four dimensions of support using simplified variables that measure the balance in each dimension (received–given). Grandparenting is a separate variable. In the adult child sample, all four dimensions of support matter. For each type of support, there are significant effects of the actual balance on the odds of being a donor (negative) and on the odds of being a receiver (positive). I also find the expected effect of grandparenting: if parents are taking care of grandchildren, children more often perceive that parents give more. In the ageing parent sample, I also find the expected effects of the four dimensions of support on the odds of being a donor. Effects are not always significant on the odds that parents are a receiver, but this group of parents is small. Although most support dimensions matter, we do see some differences in the strength of the effects. Emotional support is most closely related to the perceived degree of symmetry. This shows that the affective dimension of solidarity plays an important role in how parents and children evaluate the degree of symmetry. I also find no effect of grandparenting on how parents perceive the balance, in contrast to what was found in the child sample.

All in all, there is a good correspondence between the objective measures of balance and the subjective measure of balance. What clearly differs is that overall, many parents and children do not perceive the relationship as asymmetrical.

### Moderator Effects

To what extent are the age patterns heterogeneous? One could expect that in particular gender and education will play a moderating role, given that both factors have clear main effects on intergenerational relations. In Table 6, the parental age effects are interacted with the gender of the child, the gender of the parent, the education of the child, and the education of the parent. Models are estimated for upward support, downward support, and the balance variable. I use data from both samples (pooled). I chose a random-effects model because the main effects of gender and education are also of interest.

**Table 6:** Random-effects models with moderator variables: parent and child reports

	(1a)	(1b)	(1c)	(2a)	(2b)	(2c)
	Support to parent	Support from parent+	Up–down+	Support to parent	Support from parent+	Up–down+
Parent versus child report	− 3.003*	2.835*	− 6.052*	− 3.006*	2.826*	− 6.061*
	(.223)	(.317)	(.296)	(.224)	(.317)	(.295)
Age parent (− 70)	.221*	− .467*	.725*	.218*	− .458*	.713*
	(.011)	(.013)	(.014)	(.011)	(.013)	(.014)
Daughter versus son	1.513*	3.292*	− 1.685*	1.581*	3.268*	− 1.659*
	(.212)	(.274)	(.267)	(.214)	(.273)	(.265)
Mother versus father	3.279*	2.358*	1.017*	3.318*	2.281*	1.084*
	(.175)	(.233)	(.213)	(.177)	(.231)	(.212)
Child higher education	.611*	2.305*	− 1.790*	.704*	2.167*	− 1.575*
	(.223)	(.300)	(.290)	(.226)	(.298)	(.287)
Parent higher education	.863*	1.436*	− .536	.661*	1.569*	− .790*
	(.255)	(.355)	(.341)	(.265)	(.346)	(.338)
Age × daughter				.091*	− .184*	.272*
				(.021)	(.024)	(.026)
Age × mother				.042*	− .214*	.244*
				(.019)	(.020)	(.022)
Age × child education				.009	− .014	.050^~^
				(.022)	(.025)	(.028)
Age × parent education				− .095*	.053^~^	− .129*
				(.027)	(.031)	(.033)
Constant	23.061*	23.031*	− .036	23.027*	23.070*	− .097
	(.147)	(.185)	(.176)	(.148)	(.185)	(.176)
Dyads × waves	25,398	22,704	22,704	25,398	22,704	22,704
Dyads	8977	8955	8955	8977	8955	8955
Wald Chi-2	1098	1911	3592	1144	2073	3968

Standard errors in parentheses. Parent to child+ and up/down+ include grandparenting

^~^*p* < .10; *p < .05

*Source*: Netherlands Kinship Panel Study

Looking at the main effects first (Model 1a, 1b, and 1c), we see that mothers and daughters exchange more support. Daughters give more than sons, but they also receive more. Since the latter effect is stronger than the former, daughters are net receivers more often than sons. For parents, a similar pattern is observed. Mothers give and receive more than fathers, but they also tend to be net receivers more often. Education also has significant effects. More highly educated parents and children exchange more support than lower educated parents and children. The main effects of education are stronger for downward than for upward support. As a result, parents in more highly educated families are net donors more often than parents in lower educated families.

In the next models (Model 2a, 2b, and 2c), the parental age effects are interacted with gender and education. For all three outcome variables, I find significant interactions between parental age and the gender of the child. The support of daughters to parents increases faster with age than the support of sons to parents and the balance increases more strongly with age in daughter-parent ties. Partly similar interactions are found for the gender of the parent. Support from mothers declines more quickly with age than support from fathers and the balance increases more rapidly for mothers. Both these interactions imply that the age at reversal comes at an earlier age in mother-daughter ties. There are some significant interactions between education and parental age, but these effects are rather small in magnitude. Gender is clearly a stronger moderating factor than education.

## Conclusion

Using prospective longitudinal survey data from the Netherlands, this paper has re-examined how support exchange between parents and adult children changes as parents become older. Although there have been many longitudinal studies of adult intergenerational relations, few have explicitly isolated the effect of age. Theoretically, the age variable has played an important role, however. The classic view has been that ageing leads to a reversal in the flow of support. This view has obvious validity in many non-western societies where financial support to older parents is essential (Cong and Silverstein 2011; Agree et al. 2005; Rindfuss et al. 2012), but has been questioned for the western case. In western and especially highly developed welfare states, social exchange perspectives are often believed to be less relevant. In their place, models of parental altruism have been proposed in which parents continue to care for their children, even at very high ages.

Somewhat in contrast to these alternative views, this study shows that there is a clear reversal of the support flow. This reversal occurs for two reasons. First, parents receive more and more support from their children as they age. This points to increasing demand for support and the tendency of children to meet those demands, at least in part. Second, parents give less and less as they age. The decline in downward support and the increase in upward support lead to a crossover point at around age 75–76. This finding suggests a pattern of what we can call ‘delayed flow reversal’. It is further striking that the decline in the support that parents give is steeper than the increase in the support that parents receive. Hence, the reversal is driven more by declining opportunities for giving than by changing needs on the part of parents. In other words, we see a parent-driven flow reversal. Declining health, energy, and mobility probably plays an important role here.

Age effects were estimated in two samples: a sample of ageing parents who report about their children and a sample of adult children who report about their ageing parents. I found similar age effects in the two designs, but the strength of the effects differs considerably. Notably, the increase in upward support with age is larger when the focus is on adult children. This is due to an often observed problem of selective nonresponse of older persons (e.g. Kelfve et al. 2013), which leads to a wider parental age range and probably more ill and frail parents in the child sample. The parent data therefore reveal an age at reversal which is probably too late. There is also a tendency on both parents and children to report more frequent giving and less frequent receiving (Kim et al. 2011). This would suggest that using only the child sample is not practical either and was a reason to find the ‘truth in the middle’ by pooling the two sets of data.

A novelty is the incorporation of subjective elements in the study. In general, perceptions of symmetry follow the same dynamic pattern as the more objective measures of support exchange. Notable is that there are few parents who report that they receive more than they give. This may be due to the tendency to include affectional elements in the measure of perceived support. Alternatively, parents may include past exchanges in their evaluations of the current balance. Be that as it may, there was a good deal of correspondence between the objective measures of support exchange and the perceptions of symmetry. Grandparenting was an exception: for children, this entered the perception of symmetry but for parents it did not. Perhaps parents do not see grandparenting as pure giving to children but also as something they do for themselves. For children, grandparenting will primarily be a welcome form of support.

There are some limitations of the present study that need to be taken into account. First, I relied on an indirect way to compare different types of support. I use the contact frequencies that were associated with the different types of support rather than actual hourly investments that parents and children made. Time measures may be more accurate but are not regularly available and also do not cover more social and emotional aspects of support. Second, financial support was not included in the indices. There are data on financial transfers in the NKPS, but there is hardly any upward financial support and my main focus is on support that involves time and effort. Time and money comparisons require more complex calculations, a number of additional assumptions, and more specific time measures than the ones I have been able to use here (Litwin et al. 2008). Although several methodological challenges remain, my study does provide initial evidence for the idea of delayed and parent-driven flow reversal. For future research, it could be examined to what extent the age at reversal is similar across countries. SHARE data provide unique opportunities for estimating parental age effects based on longitudinal data in different European countries.

## Appendix

**Table 7:** Random-effects regressions for scaling support items

	(1)		(2)	
	Number of face-to-face contacts per year		Number of face-to-face contacts per year	
Household help: not	.00		.00	
Household help: incidental	4.78*	(.65)	5.94*	(1.10)
Household help: often	14.63*	(.81)	5.94*	(1.10)
Practical help: not	.00		.00	
Practical help: incidental	3.35*	(.58)	2.09*	(.97)
Practical help: often	10.50*	(.76)	11.27*	(1.27)
Emotional help: not	.00		.00	
Emotional help: incidental	10.21*	(1.09)	10.93*	(1.64)
Emotional help: often	15.88*	(1.09)	15.21*	(1.67)
Advisory help: not	.00		.00	
Advisory help: incidental	1.40*	(.64)	2.15*	(1.06)
Advisory help: often	5.10*	(.74)	7.46*	(1.24)
Grandparent help: not			.00	
Grandparent help: incidental			2.79*	(1.23)
Grandparent help: often			17.96*	(1.16)
Constant	33.02*	(1.22)	28.73*	(1.58)
Wald Chi-2	1817		1348	
Dyads × waves	51,250		22,911	
Dyads	5185		5183	

The dependent variable is the number of face-to-face contacts per year. Respondents answered on a 7-point scale (from ‘daily’ to ‘never’). Categories were recoded into the approximate number of contact times per year in days per year. The parents and children were pooled in the analysis, and the answers about giving and receiving and giving were pooled as well. Respondents are represented two times (in their role as giver and in their role as receiver). The random-effects model corrects for the fact that respondents can be represented multiple times. Model (1) is based on all observations, and Model (2) excludes observations where children report about support in an upward direction since there is no upward support with grandparenting. The effects can be seen as the approximate number of face-to-face contacts that is associated with a particular type and level of support. The scales for support are obtained by recoding the categories to the estimated effects, e.g. for household help, no help = 0, incidental = 4.8, and often = 14.6. Next, all types of support are summed, separately for giving and receiving. The estimates for grandparent help were obtained from the second model. The standard errors are in parentheses

^~^*p* < .10; **p* < .05

*Source*: Netherlands Kinship Panel Study
